# Is Objectively Measured Sitting Time Associated with Low Back Pain? A Cross-Sectional Investigation in the NOMAD study

**DOI:** 10.1371/journal.pone.0121159

**Published:** 2015-03-25

**Authors:** Nidhi Gupta, Caroline Stordal Christiansen, David M. Hallman, Mette Korshøj, Isabella Gomes Carneiro, Andreas Holtermann

**Affiliations:** 1 Department of Musculoskeletal Disorders, National Research Centre for the Working Environment, Copenhagen, Denmark; 2 Department of Occupational and Public Health Sciences, Centre for Musculoskeletal Research, University of Gävle, Gävle, Sweden; Medical University Vienna, AUSTRIA

## Abstract

**Background:**

Studies on the association between sitting time and low back pain (LBP) have found contrasting results. This may be due to the lack of objectively measured sitting time or because socioeconomic confounders were not considered in the analysis.

**Objectives:**

To investigate the association between objectively measured sitting time (daily total, and occupational and leisure-time periods) and LBP among blue-collar workers.

**Methods:**

Two-hundred-and-one blue-collar workers wore two accelerometers (GT3X+ Actigraph) for up to four consecutive working days to obtain objective measures of sitting time, estimated via Acti4 software. Workers reported their LBP intensity the past month on a scale from 0 (no pain) to 9 (worst imaginable pain) and were categorized into either low (≤5) or high (>5) LBP intensity groups. In the multivariate-adjusted binary logistic regression analysis, total sitting time, and occupational and leisure-time sitting were both modeled as continuous (hours/day) and categorical variables (i.e. low, moderate and high sitting time).

**Results:**

The multivariate logistic regression analysis showed a significant positive association between total sitting time (per hour) and high LBP intensity (odds ratio; OR=1.43, 95%CI=1.15-1.77, *P*=0.01). Similar results were obtained for leisure-time sitting (OR=1.45, 95%CI=1.10-1.91, *P*=0.01), and a similar but non-significant trend was obtained for occupational sitting time (OR=1.34, 95%CI 0.99-1.82, *P*=0.06). In the analysis on categorized sitting time, high sitting time was positively associated with high LBP for total (OR=3.31, 95%CI=1.18-9.28, *P*=0.03), leisure (OR=5.31, 95%CI=1.57-17.90, *P*=0.01), and occupational (OR=3.26, 95%CI=0.89-11.98, *P*=0.08) periods, referencing those with low sitting time.

**Conclusion:**

Sitting time is positively associated with LBP intensity among blue-collar workers. Future studies using a prospective design with objective measures of sitting time are recommended.

## Introduction

Low back pain (LBP) is a major global health problem with annual prevalence rates ranging between 22 to 65% [1]. An increasing trend of LBP from 22% in 1987 to 30% in 2005 has been observed in the general working population in Denmark [2], which is consistent with other countries [3, 4]. The direct and indirect costs of LBP are enormous due to excessive use of health services, absence from work, and lost productivity [5–8].

Adults generally spend as much as 6–8 hours per day or more than 45–50% of their waking hours in a sitting position [9–13]. Previous research has indicated that prolonged sitting may be a risk factor for developing LBP [14, 15]. Possible mechanisms mentioned for a causal relationship between prolonged sitting and LBP are increased intra-discal pressure [16], stiffness of the lumbar spine [17], reduced strength of the lower back muscles [17, 18] and/or decreased metabolic exchange leading to excessive body weight [19]. In a cross-sectional study, Omokhodion and Sanya [20] observed a significant association between self-reported occupational sitting time (i.e. for more than 3 hours) and increased severity of LBP. This finding between sitting time and LBP is in line with some cross-sectional [20–27] and prospective studies [28], while being in contrast with other studies [29–32].

One reason for these contrasting results may be that most studies have utilized self-reported measurements of sitting time [24, 33]. Self-reported measures of sedentary behavior may be biased and provide less reliable and valid estimates than objective measures [34–37]. It was, therefore, suggested that objective measures of sedentary behavior should be preferred in investigations on its association with health outcomes [38]. Another reason for the contrasting results may be that several studies [39, 40] on the association between sitting time and LBP have been conducted on heterogeneous populations with respect to socioeconomic status [29, 31, 32], which may increase the risk for socioeconomic residual confounding [41]. Also, many studies investigating the association of sitting time with LBP have been performed on study populations with a relatively low variability of sitting time, such as office workers [42]. Therefore, literature may particularly benefit from studies among groups of workers homogenous according to their socioeconomic status and with higher between-subject variability of sedentary behavior.

Moreover, only few studies have measured both occupational and leisure-time sitting to investigate their possible independent or combined association with LBP [43]. Because occupational and leisure-time sitting may be correlated [44], it is important to either perform a combined analysis of occupational and leisure-time sitting, or to mutually adjust for these variables when investigating their independent association with LBP. Another reason for separating work and leisure periods is that they might differ with respect to the temporal variation in sitting periods, which could be important to both metabolic [45] and musculoskeletal symptoms [46].

Therefore, the aim of this study was to investigate the association between objectively measured sitting time (daily total, and during occupational and leisure periods) and LBP among blue-collar workers.

## Material and Methods

### Study Population and Design

This study is a part of a cross-sectional ‘New method for Objective Measurements of physical Activity in Daily living (NOMAD)’ study in Denmark. Blue-collar workers were recruited from seven workplaces (i.e., construction workers, cleaners, garbage collectors, manufacturing workers, assembly workers, mobile plant operators and workers in the health service sector). These workplaces were selected with the purpose to investigate a large dispersion in sitting time while being relatively homogeneous with respect to socioeconomic status. The workplaces were recruited primarily through contact with trade unions or safety representatives at the individual workplaces. The inclusion criterion at workplace level was the possibility for the workers to participate in the study activities during paid working hours. Inclusion criteria for individuals to participate in the study were primary work of at least 20 hours per week, being between 18 and 65 years of age, and giving a signed informed consent. Exclusion criteria were being a white-collar worker, pregnant, having fever, and reporting skin allergy to adhesive tape used for mounting objective measurement devices.

The study was approved by the Ethics Committee for the Capital Region of Denmark (journal number H-2–2011–047) and conducted in accordance with the Helsinki declaration [47].

The reporting of the study follows the recommendations of “Strengthening the Reporting of Observational Studies in Epidemiology” statement [48].

### Procedure

All workers were invited to information meetings where the aim, contents, requirements and activities of the study were explained. All invited workers in the information meetings completed a screening questionnaire containing general information about demographic variables. Interested workers voluntarily provided their written informed consent to participate in the study. Data collection was conducted over four days with research staff visiting the workers at the workplace on days one and four. On the first day, workers interested in participating in the study (a) underwent anthropometric measurements, (b) were equipped with accelerometers for objective measures of sitting time, and (c) completed a short questionnaire containing a single item regarding LBP intensity. On day four, the workers returned the objective measurement devices.

#### Low back pain intensity

Self-reported information about LBP intensity was obtained using a modified version of the Standardized Nordic questionnaire for the analysis of musculoskeletal symptoms [49]. Workers were asked to rate their worst intensity of pain in the lower back during the past month on a response scale from 0–9, where 0 equaled ‘no pain’ and 9 equaled ‘worst imaginable pain [50]. The scale is horizontally oriented to resemble the visual analogue scale [51]. The workers were categorized into a ‘low’ (≤5), and ‘high’ (>5) pain group for the statistical analyses. These cut points were chosen based on a previous prospective cohort study on musculoskeletal risk factors for sickness absence among healthcare workers [50].

#### Objective measures of sitting time

Sitting time was estimated using two accelerometers (Actigraph GT3X+, Actigraph LLC, Florida, USA) for approximately four continuous days (4 x 24 hours)—a period generally covering at least two full working days including both occupational and leisure periods. Actigraph is a compact water resistant device (19x34x45mm, weight 19g) which measures tri-axial acceleration with a frequency of 30 Hz, a dynamic range of ±6G (1G = 9.81m/s^2^) and precision of 12 bit.

Using Fixomull (Fixomull BSN medical GmbH, Hamburg, Germany), a double side tape (3 M, Hair-Set, St. Paul, Minnesota, USA) and a waterproof film (OpSite flexifix, Smith & Nephew, London, England), two accelerometers were placed at the recommended and standardized position on the thigh and trunk [52, 53]. One accelerometer was placed at the medial front of the right thigh, midway between the hip and knee joints [52]. The other accelerometer was placed at processus spinosus at the level of T1–T2 [53, 54]. The workers were instructed (a) to take off the accelerometers if they caused itching or any kind of discomfort such as disturbed sleep, (b) to perform a reference measurement in a standing upright position for 15 sec every day, and (c) to fill in a short diary everyday concerning their working hours, leisure time, sleep, non-wear time, and time of reference measurement.

Initialization of the Actigraph for recording and downloading of data was done using the manufacturer’s program (Actilife Software version 5.5, ActiGraph LLC, Pensacola, FL, USA).

The accelerometer data were further analyzed using a specially developed MATLAB based program, Acti4 (The National Research Centre for the Working Environment, Copenhagen, Denmark and Federal Institute for Occupational Safety and Health, Berlin, Germany) estimating the type, duration and variation of physical activities and body postures with high sensitivity and specificity [52]. In short, accelerometer data are first low-pass filtered with a 5 Hz 4^th^ order Butterworth filter and then split-up into 2 sec intervals with 50% overlap. Afterwards, the individual’s reference measurement (i.e. standing in an upright position for 15 sec on every measured day) values of the thigh and trunk accelerometer were utilized to obtain the coordinate transformation between the axis of the accelerometers and the orientation of the thigh and trunk. The occurrence of sitting postures were identified according to a slightly revised procedure from Skotte, Korshoj [52] study, mainly due to the need to differentiate lying from sitting posture. In our study, sitting posture was defined as the posture in which the inclination of the thigh accelerometer is above 45° and the trunk accelerometer is below 45° [52]. In lying posture, the inclination of the thigh accelerometer is below 45° and the trunk accelerometer is above 45°.

All non-working days, sleep or bedtime periods, and non-wear periods were excluded from the analysis. The non-wear periods were identified based on the following criteria: (a) the software detected a period longer than 60 minutes showing zero counts per minute, (b) the worker reported non-wear periods, and (c) artefacts or missing data were detected by visual inspection.

Subsequently, sitting time on working days was categorized into the following settings; (a) total sitting time (i.e. total measured sitting hours divided by the number of days), (b) occupational sitting (i.e. total measured sitting hours during working periods divided by the number of days) and (c) leisure-time sitting (i.e. total measured sitting hours during leisure periods divided by the number of days). A day consisted of 24 hours starting from midnight to midnight. Work was defined as the self-reported hours spent at the primary occupation and leisure time as the remaining waking hours on a working day.

As this study was intended to address working days only, we included days comprising objective measurements of at least 4 hours of work per day. Further, only workers with at least one valid day comprising at least 10 hours of wear time were included for the analysis on total sitting time. For the specific analyses on occupational and leisure-time sitting, we included workers with at least one valid day of measurement. A valid day was based on the following criteria: (a) valid work: at least 4 hours of work time and 75% of the individual’s average reported working time, and (b) valid leisure time: at least 4 hours of leisure time and 75% of the individual’s average reported leisure time. The reason for choosing these criteria was to prevent bias due to inclusion of extreme unrepresentative data in the analysis and to reflect optimal daily wear time for valid measurements of sitting time. At least one valid day of measurement for estimating physical activities has been used as supported by previous studies [11, 55–57].

All sitting time variables were first treated as continuous variables. Later, to inspect for the possible non-linear relationship between sitting and LBP, the sitting time was also categorized into tertiles, termed as low, moderate and high sitting time.

#### Covariates

Gender was determined using a question “*are you male or female*?” The age of the workers was determined from the workers’ Danish unique civil registration number (CPR-number). Smoking behavior was determined from the question: “*Do you smoke*?*”* with 4 response categories summarized into two groups; yes (yes daily; yes sometimes) and no (used to smoke, not anymore; and I have never smoked). Seniority in the job (total months) was determined with the question: “*For how long have you had the kind of occupation that you have now*?*”* The influence at work (decision authority/latitude) of the workers was determined by the 4-item scale from the Copenhagen Psychosocial Questionnaire [58] with Cronbach’s Alpha of 0.77. A sample item is “*Do you have a large degree of influence concerning your work*?*”*. The responses were scored on a Likert scale with response categories ranging from 0 (never) to 5 (always). A composite scale measuring influence at work was constructed by calculating the mean rating of all four items. For the analysis, this scale was recoded to 0–100 scale, whereby a larger score represented a higher degree of influence at work. The body mass index (BMI, kg/m^2^) was calculated using objectively measured height (m) and body weight (kg). The self-reported information on total time spent on occupational carrying and lifting was collected using a question: *How much of your working time do you spend carrying or lifting things*? with six response categories summarized into three groups; high lifting/carrying time (almost all the time, approximately ¾ of the time, and ½ of the time), moderate lifting/carrying time (¼ of the time) and low lifting/carrying time (rarely/very little and never). Age, job seniority, BMI, and influence at work were treated as continuous variables, while time spent carrying/lifting at work, gender, and smoking were entered as categorical variables in the statistical analyses.

### Statistical Analyses

The association between total sitting time and LBP intensity was determined using binary logistic regression. In the primary model, dichotomized LBP [categorized into *low* (≤5) and *high* LBP (>5)] was entered as a dependent variable and continuous measures of total sitting time (in hours) as an independent variable. The model was further step-wise adjusted with the following covariates; step 1) age, gender, BMI, and smoking, step 2) step 1 + job seniority, influence at work, and occupational lifting/carrying time at work. Similar analyses were also performed by regressing the high and moderate sitting time categories against high LBP, referencing the low sitting time category.

Moreover, similar logistic regression analyses were performed on continuous (in hours) and categorical measures of occupational and leisure-time sitting (i.e. low, moderate and high), while adjusting for the above-mentioned covariates. Additionally, in the final step, the model was further adjusted for occupational or leisure-time sitting depending on the modelled variable (Step 3).

To test the consistency of the results, four different sensitivity analyses were performed. First, a similar logistic model (as explained above) was performed using a lower and higher cut point of high LBP intensity (i.e. >4 on the 0–9 scale and i.e. >6 on the 0–9 scale) than the cut point used in the primary analysis (i.e. >5 on the 0–9 scale). Second, further logistic regression analyses were performed modeling the normalized sitting time based on average measured hours per day (i.e. percent total sitting) instead of using absolute time of sitting as in the primary analysis. Third, similar analyses were performed using log binomial regressions to calculate the relative risk (RR) instead of OR calculated via the logistic regression. Fourth, to investigate a potential bias due to wear time on the association between sitting time and LBP, we performed similar logistic regression analyses as in the primary analysis with further adjustment for number of valid days measured or average measured time per day.

Multi-collinearity diagnostic indicated no multi-collinearity issues (condition indices <30 and VIF values <10) between the chosen independent variables in this study. The covariates were selected *a priori* based on previous studies on risk factors of LBP [59–69]. Specifically age, gender, BMI, and smoking were identified as potential individual risk factors while occupational lifting/carrying time, influence at work, and job seniority as occupational risk factors.

In all models, the OR/RR with 95% confidence intervals (CI) was estimated indicating the probability of reporting high LBP. Statistical Package for the Social Sciences (SPSS, IBM, version 21.0) was utilized to perform all statistical operations. The level of significance was set at *P*<0.05.

## Results

### Characteristics of the Study Population

**Fig 1:**
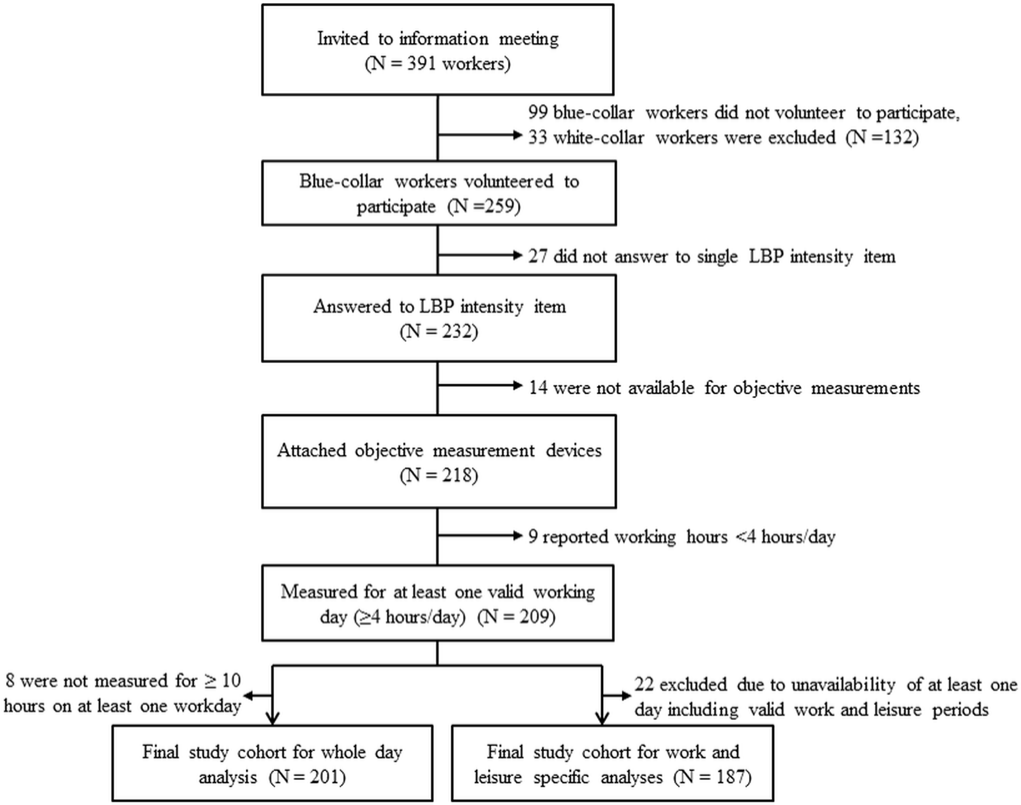
Flow diagram of the study population of blue-collar workers.

Out of 391 workers who were invited to the information meeting, 358 (92%) workers were engaged in blue-collar occupations and being eligible for the study. Out of them, 259 (66%) volunteered to participate in our study and 218 of them (56%) answered to the single item of LBP (pain intensity) and participated in the objective measurements. Nine workers were excluded due to unavailability of at least one valid work day measurement. Eight out of 209 from the analysis on total sitting time and twenty-two out of 209 workers from the specific analyses on occupational and leisure-time sitting were excluded due to unavailability of at least one day with valid objective measurement periods. Finally, 201 (51%) and 187 (48%) workers were included in the main analyses on total, occupational and leisure-time sitting, respectively (Fig. 1).

In the analysis on total sitting time, a total of 8,223 valid waking hours were included with 500 valid days and a mean (SD) of 16.6 (1.6) waking hours per day. On average, workers were measured for 2.5 (SD 1.1) days, and 80% of the workers wore accelerometers for ≥2 valid measured days. In the occupational and leisure-time specific analyses, a total of valid 3,212 occupational and 3,151 leisure hours over 368 days, with 8.9 (SD 2.6) leisure, and 8.4 (SD 2.4) occupational hours per day were included.

On average, the participating blue-collar workers sat for 7.3 (SD 2.1) hours of total time, 3.0 (SD 1.4) hours of work time and 4.8 (SD 1.8) hours of leisure time each day. Total, occupational and leisure-time sitting ranged between 2.5–13.5, 0.3–6.6, and 0.7–10.3 hours, respectively.

A significant amount of workers (28%) were manufacturing workers, followed by assemblers (16%), cleaners (15%) and construction workers (18%), while the remaining workers were garbage collectors (9%), personal care workers (7%) and drivers (5%). Sixteen percent of the workers reported high LBP (>5). The participating workers were predominantly males (58.2%), non-smokers (56.8%), had low influence at work (mean 43.7, SD 23.3) and reported moderate to high occupational lifting and carrying time (66.5%) (Table 1).

**Table 1:** Demographic characteristics of the total blue-collar workers and stratified on their low back pain (LBP) intensity [low LBP ≤5, high LBP >5 on a scale from 0 (no pain) to 9 (worst imaginable pain)].

**Variables**	**Total**	**Low LBP**	**High LBP**
n	201	168	33
**Gender**			
Male, n (%)	171 (58.2)	97 (57.7)	20 (60.6)
Female, n (%)	84 (41.8)	71 (42.3)	13 (39.4)
Age in years, M (SD)	44.7 (9.7)	44.9 (9.8)	43.7 (9.4)
Seniority in months, M (SD)	160.3 (135.6)	161.2 (131.8)	155.8 (156.3)
BMI in kg/m^2^, M (SD)	26.4 (5.0)	26.3 (5.1)	26.6 (5.0)
Influence at work in 0–100%, M (SD)	43.7 (23.3)	43.8 (22.9)	43.0 (25.6)
Mean hours measured per day, M (SD)	16.6 (1.6)	16.5 (1.5)	17.1 (1.8)
Smokers, n (%)	82 (43.2)	67 (42.1)	15 (48.4)
**Occupational lifting/carrying time, n (%)**			
Low	67 (33.5)	53 (31.7)	14 (42.4)
Moderate	50 (25.0)	42 (25.1)	8 (24.2)
High	83 (41.5)	72 (43.1)	11 (33.3)
**Total sitting time in hours, M (SD)**	7.3 (2.1)	7.0 (2.0)	8.6 (2.3)
Low, n (%)	67 (33.3)	60 (35.7)	7 (21.2)
Moderate, n (%)	67 (33.3)	60 (35.7)	7 (21.2)
High, n (%)	67 (33.3)	48 (28.6)	19 (57.6)
**Valid days of measurements, M (SD)**	2.5 (1.1)	2.5 (1.1)	2.31.1
Valid 1-day measurement, n (%)	201 (100)	168 (100)	33 (100)
Valid 2-day measurement, n (%)	160 (79.6)	135 (80.4)	25 (75.8)
Valid 3-day measurement, n (%)	96 (47.8)	85 (50.6)	11 (33.3)
Valid 4-day measurement, n (%)	40 (19.9)	35 (20.8)	5 (15.2)
Valid 5-day measurement, n (%)	3 (1.5)	2 (1.2)	1 (3.0)

Table footnotes:

BMI = body mass index, LBP = low back pain, M = mean, n = number of workers, SD = standard deviation.

Table 1 shows the characteristics of the included workers categorized into low and high intensity of LBP. There were no marked differences between groups with high and low LBP intensity in terms of age, BMI, job seniority, influence at work, distribution of gender, and number of valid measured days. However, workers with high LBP, compared to those with low LBP, were measured for slightly longer time and were exposed to longer total sitting time. A substantial proportion (58%) of workers with high LBP were exposed to high total sitting time (i.e. >8 hours per day). There was no marked difference between the blue-collar occupations with respect to LBP intensity (F = 1.48, *P* = 0.17).

Approximately similar distributions of characteristics between low and high LBP intensity groups were observed among the 187 workers included in the occupational and leisure-time specific analyses (data not shown).

Table 2 shows the characteristics of the 201 workers stratified into low (≤6.4 h), moderate (6.5–8.3 h) and high (>8.3 h) total sitting time. The workers in the high sitting category were predominantly construction workers (28%) and assemblers (25%) while workers in the low sitting category were mostly manufacturing workers (61%). In the moderate sitting category, most workers were cleaners (21%), construction workers (19%), and manufacturing workers (18%).

**Table 2:** Demographic characteristics of the blue-collar workers stratified on their objectively measured total sitting time.

Variables	Low	Moderate	High
n	67	67	67
**Gender**			
Male, n (%)	36 (53.7)	40 (59.7)	41 (61.2)
Female, n (%)	31 (46.3)	27 (40.3)	26 (38.9)
Age in years, M (SD)	43.7 (9.6)	45.4 (10.7)	45.0 (8.9)
Seniority in months, M (SD)	145.4 (137.9)	164.3 (126.4)	171.1 (142.8)
BMI in kg/m^2^, M (SD)	25.7 (4.2)	26.0 (4.8)	27.4 (5.9)
Influence at work in 0–100%, M (SD)	38.8 (22.5)	42.9 (21.7)	49.3 (24.7)
Mean hours measured per day, M (SD)	16.0 (1.5)	16.6 (1.3)	17.1 (1.7)
Smokers (%)	26 (40.6)	27 (42.9)	29 (46.0)
**Occupational lifting/carrying time, n (%)**			
Low	23 (34.3)	23 (34.8)	21 (31.3)
Moderate	16 (23.9)	15 (22.7)	19 (28.4)
High	28 (41.8)	28 (42.4)	27 (40.3)
**LBP pain, M (SD)**	2.7 (2.4)	2.4 (2.5)	3.6 (2.7)
Low, n (%)	60 (89.6)	60 (89.6)	48 (71.6)
High, n (%)	7 (10.4)	7 (10.4)	19 (28.4)
**Valid days of measurements, M (SD)**	2.7 (1.1)	2.4 (1.0)	2.4 (1.1)
Valid 1-day measurement, n (%)	67 (100)	67 (100)	67 (100)
Valid 2-day measurement, n (%)	57 (85.1)	53 (79.2)	50 (74.7)
Valid 3-day measurement, n (%)	37 (55.3)	28 (41.8)	31 (46.3)
Valid 4- day measurement, n (%)	18 (26.9)	10 (15)	12 (18)
Valid 5- day measurement, n (%)	2 (3.0)	1 (1.5)	0 (0.0)

Table footnotes:

Low sitting = ≤6.4h, moderate sitting = 6.5–8.3h and high sitting >8.3h, BMI = body mass index, LBP = low back pain, M = mean, n = number of workers, SD = standard deviation.

The workers with high total sitting time were similar to the workers with low sitting time in most characteristics except that they were measured for slightly longer time, had slightly higher influence at work and more reported high LBP. The workers with moderate and low sitting time were approximately similar in terms of their overall descriptive characteristics.

When categorized based on occupational sitting time, the workers with high (>3.7h) and moderate sitting time (2.1–3.7h) were not significantly different from those with low sitting time (≤2.0h) for most descriptive characteristics, except for LBP intensity.

When categorized based on leisure-time sitting, the workers with high sitting time (>5.4h) were similar to those with low sitting time (0–3.9h), except that workers with high sitting time had higher job seniority, BMI, influence at work and LBP. However, no pronounced differences were observed between workers with low and moderate sitting time (4.0–5.4h).

### Association between the continuous measures of sitting time and LBP intensity

Table 3 presents the results of the association between hours of sitting and high LBP intensity. There was a significant positive association between total sitting time and high LBP intensity (OR = 1.43). Adjustment for various confounders such as individual factors (age, gender, BMI, and smoking) and occupational factors (seniority, influence at work, and occupational lifting/carrying time) did not markedly influence the estimates or level of significance. We observed similar results for leisure-time sitting and high LBP intensity (OR = 1.38), which remained consistent after additional adjustment for occupational sitting time. We also found positive associations between occupational sitting time and high LBP intensity (OR = 1.36), but although the estimates remained similar, they became marginally insignificant (P = 0.06) with further adjustment for individual and occupational factors.

**Table 3:** Binary logistic regression analyses of the association between objectively measured sitting (hours/day) and high LBP intensity (>5 on scale 0–9) among blue-collar workers.

	Total sitting time				Occupational sitting time				Leisure-time sitting			
**Model**	n	OR	P	95%CI	n	OR	P	95%CI	n	OR	P	95%CI
**Crude** ^a^	201	1.43	0.01	(1.17–1.73)	187	1.36	0.03	(1.03–1.80)	187	1.38	0.01	(1.10–1.71)
**Step 1** ^b^	190	1.41	0.01	(1.15–1.73)	176	1.31	0.07	(0.98–1.75)	176	1.41	0.01	(1.10–1.82)
**Step 2** ^c^	185	1.43	0.01	(1.15–1.77)	172	1.30	0.08	(0.97–1.75)	172	1.41	0.01	(1.08–1.84)
**Step 3** ^d^	-	-	-	-	172	1.34	0.06	(0.99–1.82)	172	1.45	0.01	(1.10–1.91)

Table footnotes:

n = number of workers, OR = odds ratio, 95%CI = 95% confidence interval,

^a^ Crude model,

^b^ Adjusted for age, gender, smoking and BMI (Individual factors, Step 1).

^c^ Step 1+seniority, influence at work, and occupational lifting/carrying time (occupational factors, step 2).

^d^ Step 2+sitting time in the opposite domain (Step 3).

### Associations between categories of total, occupational, and leisure-time sitting with LBP intensity

Table 4 shows the results of the binary logistic regression analyses on the association between categories of sitting time and high LBP intensity, with low sitting time as reference. The workers with high sitting time had a significantly higher probability of reporting high LBP intensity, both for total and leisure-time sitting. Adjustment for various confounders imposed no marked effect on these estimates or levels of significance. Reduced and non-significant positive associations were observed for moderate sitting for total (OR = 1.13) and leisure (OR = 1.39) time and high LBP. For the analysis based on categories of occupational sitting, we obtained positive and significant results in the crude model for moderate (OR = 4.53) and high (OR = 3.48) sitting time and high LBP. These estimates were not reduced with further adjustment for potential individual and occupational confounders, although the estimates for high sitting became marginally non-significant (*P* = 0.08) (Table 4).

**Table 4:** Results of binary logistic regression models estimating the association between categories of sitting time and high intensity of low back pain (>5 on a scale 0–9) among blue-collar workers.

Model	Total sitting time				Occupational sitting time				Leisure-time sitting			
	n	OR	P	95%CI	n	OR	P	95%CI	n	OR	P	95%CI
**Crude** ^**b**^												
Low		1^a^				1^a^				1^a^		
Moderate	201	1.00	1.00	(0.33–3.03)	187	4.53	0.01	(1.41–14.56)	187	1.66	0.40	(0.51–5.38)
High		3.39	0.02	(1.32–8.74)		3.48	0.04	(1.06–11.47)		4.67	0.01	(1.61–13.54)
**Step 1** ^**c**^												
Low		1^a^				1^a^				1^a^		
Moderate	190	1.08	0.90	(0.35–3.30)	176	4.82	0.01	(1.46–15.96)	176	1.47	0.54	(0.43–5.03)
High		3.21	0.02	(1.20–8.60)		2.86	0.10	(0.83–9.83)		5.34	0.01	(1.71–16.66)
**Step 2** ^**d**^												
Low		1^a^				1^a^				1^a^		
Moderate	185	1.13	0.84	(0.36–3.52)	172	4.49	0.01	(1.34–15.06)	172	1.50	0.52	(0.43–5.20)
High		3.31	0.03	(1.18–9.28)		2.88	0.10	(0.83–10.05)		5.06	0.01	(1.57–16.34)
**Step 3** ^**e**^												
Low	-	-	-	-		1^a^				1^a^		
Moderate	-	-	-	-	172	4.89	0.01	(1.40–17.10)	172	1.39	0.61	(0.39–4.92)
High	-	-	-	-		3.26	0.08	(0.89–11.98)		5.31	0.01	(1.57–17.90)

Table footnotes:

Total sitting time: low ≤6.4h, moderate = 6.5–8.3h and high >8.3h; Occupational sitting: low ≤2.0h, moderate = 2.1–3.7h and high>3.7h; leisure-time sitting: low≤3.9h, moderate = 4.0–5.4h and high>5.4h, n = number of workers, OR = odds ratio, 95%CI = 95% confidence interval,

^a^ Reference

^b^ Crude analysis,

^c^ Adjusted for age, gender, smoking and BMI (individual factors, step 1).

^d^ Step 1 + seniority, influence at work and occupational lifting/carrying time (occupational factors, step 2).

^e^ Step 2 + sitting time during opposite domain depending on the modelled variable (step 3)

### Sensitivity analyses

To test the robustness of the results from the primary analyses, four additional sensitivity analyses were performed;

a) Logistic regression models on the association between hours of sitting and LBP were performed using lower (>4 on the 0–9 scale) or higher (>6 on the 0–9 scale) cut-points of high LBP intensity than the cut point used in the primary analysis (>5 on the 0–9 scale). In the analysis using a lower cut-point of high LBP, we obtained slightly reduced but significant positive associations for total sitting time (OR = 1.20, 95%CI = 1.01–1.42) and reduced, non-significant tendencies for a positive association for leisure-time sitting (OR = 1.17, 95%CI = 0.93–1.47) and occupational sitting (OR = 1.30, 95%CI = 0.99–1.68) compared to the primary analyses. When using a higher cut-point of LBP, positive tendencies were found, but the ORs were reduced and did not remain significant (total sitting time, OR = 1.21, 95%CI = 0.95–1.53; occupational sitting, OR = 1.24, 95%CI = 0.87–1.75; and leisure-time sitting, OR = 1.17, 95%CI = 0.85–1.61). For categorized sitting time, changing the cut-point of high LBP resulted in reduced non-significant positive associations between sitting time and LBP, both for total and occupational sitting, while remaining significant for leisure-time sitting when using the lower cut-point of LBP (data not shown).

b) Similar analyses were performed using normalized sitting time based on mean measured hours per day (i.e., percent sitting) instead of using absolute sitting time as in the primary analysis. Results showed reduced positive associations compared to the primary analysis [total sitting, OR = 1.05 (95%CI = 1.01–1.09), occupational sitting, OR = 1.04 (95%CI = 0.99–1.09) and leisure-time sitting, OR = 1.06 (95%CI = 1.01–1.12)]. Similar results to the primary analysis were observed when we modelled the moderate and high categories of percent sitting time against high LBP, referencing low sitting time for total and occupational sitting periods, although the positive association was reduced and non-significant for leisure-time sitting (data not shown).

c) We performed a third sensitivity analysis to investigate if calculation of relative risks (RR) will provide similar results compared to OR used in the primary analysis. The results remained largely unchanged compared to the primary analysis. In the analysis modelling the hours of sitting against high LBP, the RR were 1.35 (95%CI = 1.15–1.58) for total sitting time, 1.35 (95%CI = 1.10–166) for leisure-time sitting and 1.26 (95%CI = 1.00–1.57) for occupational sitting time. Using the categories of sitting, the RR for high and moderate sitting time were 2.72 (95%CI = 1.17–6.32) and 1.08 (95%CI = 0.40–2.91) for total sitting time, 3.96 (95%CI = 1.50–10.46) and 1.41 (95%CI = 0.47–4.19) for leisure-time sitting and 2.80 (95%CI = 0.93–8.45) and 4.31 (95%CI = 1.43–13.00) for occupational sitting.

d) As the wear time varied between workers, we performed a fourth sensitivity analysis to investigate if estimates of the association between sitting time and LBP change with further adjustment for wear time (i.e. number of valid measured days or average measured time per day). These adjustments did not materially change the estimates, compared to the primary analysis. In the analysis with adjustment for number of valid measured days, the OR was 1.42 (95%CI = 1.14–1.76) for total sitting time, 1.35 (1.00–1.83) for occupational sitting and 1.43 (1.01–1.88) for leisure-time sitting. Similarly, with adjustment for average measured time per day, the OR was 1.35 (1.08–1.69) for total sitting time, 1.37 (1.01–1.85) for occupational sitting time, and 1.33 (0.98–1.81) for leisure-time sitting. Similar positive trends as in the primary analyses were observed when modelling categories of sitting time (low, moderate and high) against LBP (data not shown).

## Discussion

This study aimed at investigating the association between sitting time, measured objectively using diurnal accelerometry, and LBP intensity among blue-collar workers. We observed a positive association between hours of total, occupational, and leisure-time sitting and LBP intensity. The estimates remained largely unchanged after adjustment for various individual and work-related factors. These positive associations between sitting time and LBP were also confirmed in the analyses based on categories of sitting time.

Most previous studies [40, 70, 71] have only focused on investigating the association between occupational sitting time and LBP, while very few studies [72, 73] have been conducted on total sitting time. We observed a clear positive association between total sitting time and LBP intensity, even after adjusting for potential individual and work-related confounders. These results were also confirmed in the analysis using categories of sitting time. In contrast to the results of our study, a previous case-control study [73] did not observe a clear positive association between total sitting time and LBP. Levangie [73] observed that clinical outpatients reporting sitting for 4–6 hours or for more than 9 hours per day tended to report high LBP (4–6 hours: OR = 1.54, 95%CI = 0.81–2.91; >9 hours: OR. 1.42, 95%CI = 0.73–2.78), compared to those sitting for less than 4 hours per day. However, none of these results were statistically significant. Also, patients sitting for 6–8 hours per day reported lower intensity of LBP compared to those sitting less than 4 hours a day, indicating an inverse relationship between sitting and LBP. The reasons behind the inconsistent findings in previous studies could be that they utilized self-reports of sitting time that may be imprecise [35, 74, 75], and potentially biased by factors like musculoskeletal complaints [76]. This methodological aspect of previous studies may have diluted the true association between sitting and LBP in these studies. Another reason could be that previous studies did not control for various individual and work-related factors, which may have confounded the results. To our knowledge, our study is the first of its kind in using objective measures of sitting time. We found a clear positive association between total sitting time and LBP intensity, which is in contrast to some previous studies using self-reported sitting time. Thus, further studies using objective measures of sitting time are needed before a conclusion about the association between sitting and LBP intensity can be drawn.

### Association between occupational and leisure-time sitting and LBP intensity

We found a significant positive association between leisure-time sitting and LBP, even after adjusting for various potential individual and work-related confounders. A similar clear association was observed when we analyzed the association between categories (low, moderate, high) of leisure-time sitting and LBP. These results are consistent with some previous studies finding a significant positive association between sedentary time during leisure and LBP [77, 78]. However, most previous studies did not find significant or clear associations [28, 79–81]. In some studies, researchers even found tendencies for a protective effect for time spent watching TV or playing computer games on LBP [28]. Thus, future studies are needed to confirm our findings using objective measures of sitting time during leisure.

In the crude model, we observed a significant positive association between occupational sitting time and LBP. With further adjustment for different confounders, the OR did not decrease, but the association became marginally insignificant (*P* = 0.06). Similar findings were found for categories of occupational sitting time and LBP. Correspondingly, Lis, Black [40] in a meta-analysis summarized the OR for the association between occupational sitting and LBP from previous studies to be 1.99. However, half of the studies included in this meta-analysis were not significant. Although some previous studies also have found a positive association between occupational sitting time and LBP [21, 28, 82, 83], others have not [29, 30, 84].

There can be a number of reasons behind these contrasting findings of previous studies investigating the association of occupational and leisure-time sitting with LBP. First, most studies did not adjust for several potential confounders as well as for sitting time in the remaining time of the day. Second, most previous studies have relied on self-reports of sitting time, that may be inaccurate and biased [35, 76] and consequently this may have diluted a true association between sitting time and LBP. Thus, future studies using objective measures of sitting while adjusting for important confounders are needed to further confirm the findings of the present study.

### Methodological considerations, strengths and weaknesses

We hypothesized sitting to be associated with LBP. However, since the etiology of LBP is multifactorial, it was important to adjust for potential confounders in the association between sitting time and LBP. When we adjusted for a number of potential individual confounders, the results remained stable from the crude model. Additionally, the results remained consistent even after adjusting for job specific characteristics such as “influence at work” and “occupational lifting/carrying time”.

The OR tends to overestimate the magnitude of relative risk (RR) if the prevalence of the outcome is high [85]. In this study, the prevalence of the outcome ‘high LBP’ was relatively high (16%). Therefore, we also performed a sensitivity analysis using the RR instead of OR as an estimate of the association between sitting and LBP. As expected, we obtained similar trends using RR compared with the OR. These results indicate the robustness of the primary results of this study.

It has been argued that transforming continuous data into categories ought to be avoided as it results in a loss of information and reduces the precision of the individual exposure estimates [86]. On the other hand, categorizing exposure into simple categories provide useful and easily interpretable information. Thus, we performed analyses based on both continuous and categorical measures of sitting time. Both analyses generally produced similar results in their association with high LBP, which further supports the robustness of the present findings. However, using continuous percent sitting time (percentage of average measured time per day), instead of absolute sitting time as applied in the primary analyses, reduced the ORs for all analyses, although the ORs remained significant for total and leisure-time sitting. Using categories based on percent sitting time, the ORs were similar to the primary analysis besides for leisure-time sitting which was reduced and non-significant. Generally, the results of these sensitivity analyses for occupational siting remained positive but reduced and non-significant. The reduced and non-significant results may be explained by the wide range of time spent in different periods, i.e., workers being classified in different sitting categories or different extremes of the range of sitting time when using absolute values and percentages of sitting time. However, adjustment for average measured time per day in the analysis did only marginally change the estimates of the association between sitting and LBP for all domains, compared to the primary analysis.

We used a cut point of > 5 for high LBP on a scale of 0–9 which was based on a previous prospective study on the association between LBP and sickness absence [50]. However, some previous studies have used different cut-points of high LBP [87]. Thus, we tested the association between sitting time and LBP using both lower and higher cut-points of high LBP than in the primary analyses. Although, the direction of the association did not change, we obtained lower estimates for the association between total, occupational and leisure-time sitting and LBP both when applying lower and higher cut-points for LBP compared to primary analysis. We do not know the reasons for the reduced estimates and level of significance when applying lower and high cut-points for high LBP. However, it highlights the need for replicating the analyses of this study in other and larger datasets.

The main strength of this study was the use of objective measures of sitting time to determine the association of sitting time with LBP. The Actigraph accelerometers used in this study are water resistant and easy to wear during long-term measurements. This measurement device has been utilised previously on workers with physically demanding jobs [88]. Another strength of the study is the use of a validated software, Acti4, to determine the accurate sitting time. Acti4 has previously determined sitting postures during free living conditions with a high sensitivity and specificity of 98% and 93%, respectively. Additionally, we recruited a study population with a wide range of occupational and leisure-time sitting, providing a necessary contrast in the sitting time exposure despite their homogenous socioeconomic status.

The main limitation of the study is the use of a cross-sectional study design from which an inference about a causal relationship cannot be made. Thus, prospective studies on the association between objectively measured sitting time and LBP are needed. Another limitation of the study is the relatively limited sample size, which may have reduced the generalizability of our findings. Thus, future studies investigating the association between sitting time and LBP are recommended to use larger and representative sample size.

### Practical implications

The results of this study indicate a positive association between sitting time and LBP among blue-collar workers. Although, this relationship should be further investigated using a prospective design before recommendations can be made, the results of this study imply that future interventions may be needed for reducing sitting time even among blue-collar workers with high sitting exposure and LBP.

## Conclusion

To our knowledge, this is the first study to investigate the association between sitting time, assessed objectively for several days, and LBP intensity. Our study indicates that sitting time is positively associated with LBP intensity among blue-collar workers. Future studies using prospective designs with larger sample sizes and objective measurements of sitting time are needed to confirm these findings.

## Supporting Information

S1 Dataset This dataset contains two data files, ‘*total sit*’ and ‘*occupational and leisure sit*’. The data file ‘total sit’ contains data for investigating the association between total sitting time (N = 201) and low back pain and the data file ‘occupational and leisure sit’ contains data for investigating the association between occupational and leisure-time sitting and low back pain (N = 187).
(XLSX)
